# A Meteorological Table

**Published:** 1804-11-01

**Authors:** 

**Affiliations:** Brompton


					[ 480 ]
A Meteorological Table,
by Dr. Higgins, of Brompton.*
n.
20i
21
22|
23
24
25
z6\
27
28
29
3o
1
2
3
4
5
6
7
8
9
10
11
12
13
14
15
16
17
j8
Height of the Bavo-
ir.eter. Inches.
p-s>
?Z
WEATHER,
30.16,30.16,30.16
19I .21
2 9.96,2 9.84
30.13i30.19
?3i
.06
.24:
?Si
.86
? 68
.64:
?57
.26
9.96
30.04
?9
07
?27
.64
?87
.63
?63;
?47
.18
29.92
30.04
.01
05
-34|
.71
?83
?64
29.64 29.72
33.44j30.39
.25! .12
19.8729.90
30.06j30.18
?43
29.61
?47
62
?38
?3i
.82
?93
30*21
"25
?34
29.51
?39
.64
?38
?32
.87
.89
30.2c
.64 31
?34|
.02
29.86
30.12
.02
29.83
30.16
37
2977
.86
30.36
.05
29.46
?4?
?58
? 17
.56
30.02
29.98
30.24
.07
33
30
Fir.
Cloudy.
Cloudy.
Fa'r.
Windy, with (howers.
jS!iowery.
Cloudy.
Cloudy, with rain at night.
Cloudy.
Cloudy, with flight rain;
Showery, rain at night
Cloudy, with flight rain.
Cloudy, rain in the night.
Cloudy, with flight rain in the morning.
Fait, rain and wind at night.
Fair.
Fair.
Rain, ftormy at night.
Fair.
Fair.
Fair till noon, then continued rain,
Rain.
Showery, with hail.
Cloudy, rain at night.
Cloudy, rain in the evening.
Cloudy, fair in the evening.
Fair.
Showery, with a thunder ftorm.
Cloudy, with flight rain.
CI udy.
* Agreeably to the request of the Editors, I have made the necessary
observations; and find that my house is situated as nearly as possible W.
by S. from St. Paul's, and that its elevation above the high water mark at
Chelsea, is not more than 12 or 15 feet;
I have added an additional column to the present month's table, shewing
the degrees of evaporation by an hygrometer, constructed nearly upon the
principle of Mr. Leslie's; and next month, if agreeable to the Editors, I
propose giving the degrees of heat by the Centigrade as well as Fahrenheit's
thermometer, for the purpose of shewing the simplicity of the one, when
compared with the other.

				

